# Prompt Electrodeposition of Ni Nanodots on Ni Foam to Construct a High-Performance Water-Splitting Electrode: Efficient, Scalable, and Recyclable

**DOI:** 10.1007/s40820-019-0269-x

**Published:** 2019-05-16

**Authors:** Hongtao Yu, Ting Quan, Shilin Mei, Zdravko Kochovski, Wei Huang, Hong Meng, Yan Lu

**Affiliations:** 1 Soft Matter and Functional Materials, Helmholtz-Zentrum Berlin für Materialien und Energie, Hahn-Meitner Platz 1, 14109 Berlin, Germany; 2 Key Lab for Flexible Electronics & Institute of Advanced Materials, Jiangsu National Synergistic Innovation Center for Advanced Materials (SICAM), Nanjing Tech University, 30 South Puzhu Road, Nanjing, People’s Republic of China; 3 Institute of Chemistry, University of Potsdam, 14467 Potsdam, Germany

**Keywords:** Electrodeposition, Ni nanodots, Bifunctional catalysts, Water splitting, Large-size

## Abstract

In past decades, Ni-based catalytic materials and electrodes have been intensively explored as low-cost hydrogen evolution reaction (HER) and oxygen evolution reaction (OER) catalysts for water splitting. With increasing demands for Ni worldwide, simplifying the fabrication process, increasing Ni recycling, and reducing waste are tangible sustainability goals. Here, binder-free, heteroatom-free, and recyclable Ni-based bifunctional catalytic electrodes were fabricated via a one-step quick electrodeposition method. Typically, active Ni nanodot (NiND) clusters are electrodeposited on Ni foam (NF) in Ni(NO_3_)_2_ acetonitrile solution. After drying in air, NiO/NiND composites are obtained, leading to a binder-free and heteroatom-free NiO/NiNDs@NF catalytic electrode. The electrode shows high efficiency and long-term stability for catalyzing hydrogen and oxygen evolution reactions at low overpotentials (^10^*η*_HER_ = 119 mV and ^50^*η*_OER_ = 360 mV) and can promote water catalysis at 1.70 V@10 mA cm^−2^. More importantly, the recovery of raw materials (NF and Ni(NO_3_)_2_) is quite easy because of the solubility of NiO/NiNDs composites in acid solution for recycling the electrodes. Additionally, a large-sized (*S* ~ 70 cm^2^) NiO/NiNDs@NF catalytic electrode with high durability has also been constructed. This method provides a simple and fast technology to construct high-performance, low-cost, and environmentally friendly Ni-based bifunctional electrocatalytic electrodes for water splitting.

**Electronic supplementary material:**

The online version of this article (10.1007/s40820-019-0269-x) contains supplementary material, which is available to authorized users.

## Highlights

- Facile electrodeposition for fabricating active Ni nanodots (NiNDs) on Ni foam (NF) is shown.
- Binder- and heteroatom-free recyclable NiO/NiNDs@NF electrodes are efficiently made.
- NiO/NiNDs@NF bifunctional catalytic electrodes are used for water splitting.

**Electronic supplementary material:**

The online version of this article (10.1007/s40820-019-0269-x) contains supplementary material, which is available to authorized users.

## Graphical abstract

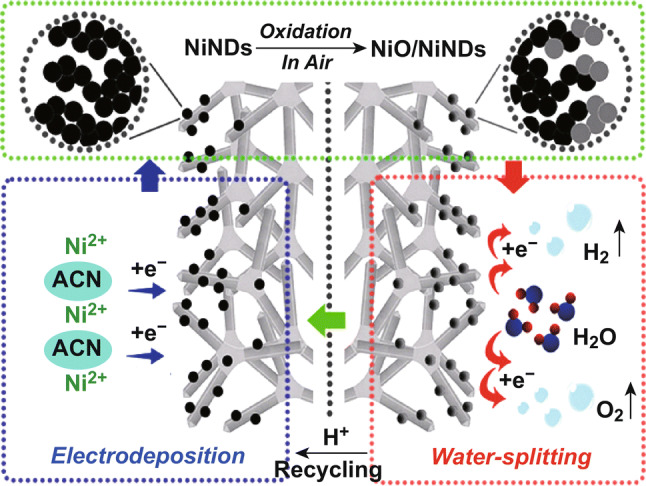

## Introduction

Growing concerns regarding the energy crisis and environmental pollution prompts the exploration of sustainable energy sources as substitutes for traditional fossil fuels [1, 2]. As an environmentally friendly energy carrier, molecular hydrogen (H_2_) plays a critical role in sustainable energy systems [3, 4]. Among the technologies for H_2_ production, the electrocatalytic H_2_ evolution reaction (HER) from water splitting is the most effective and economical route because of its high energy-conversion efficiency and environmentally benign process [5–7]. It has been confirmed that precious platinum (Pt)-based materials play a leading role in current H_2_-production technology, such as water-alkali electrolysis [8, 9]. However, the scarcity and high cost of Pt severely hamper its large-scale industrial applications. Therefore, it is crucial to explore inexpensive, alternative electrocatalysts that are made from earth-rich elements and have good activity and durability. Furthermore, water electrolysis should be carried out in either strongly acidic or alkaline electrolyte to minimize the overpotentials in the electrolyte [10]. Hence, development of a bifunctional catalyst that is based on earth-rich elements and has high activity for both HER and oxygen evolution reaction (OER) in the same electrolyte is essential for simplifying the system and reducing manufacturing costs of H_2_.

In the past decades, a number of non-noble HER electrocatalysts based on transition metals and their compounds have been explored. Among the transition metals explored, Ni atoms possess an appropriate hydrogen surface adsorption energy, which makes them broadly recognized as excellent water dissociation centers [11, 12]. However, during the catalytic process on a Ni surface, the adsorption sites for H atoms may be occupied by OH^−^ species. This causes a decrease in the active sites, which leads to a dramatic decline of catalytic activity. Ni foam (NF) is a low-cost and three-dimensional porous structure that is commonly used as the electrodes of alkaline electrolyzers for commercial applications [13]. Nanostructuring and surface engineering research has aimed to improve the catalytic performance of NF through maximizing the number of catalytic active sites and promoting mass transport. Different types of Ni-based materials (including metallic Ni, Ni-based alloys, oxides, nitrides, phosphides, and sulfides) coated on NF have been intensively studied as HER and OER catalysts for water splitting [14–23]. Despite the largely improved catalytic performance, the process for large-scale preparation and application of these catalysts is less impressive and satisfactory. Moreover, the introduction of heteroatoms (e.g., N, S, P, and Se) is not beneficial for the recovery and recycling of the electrode. With increasing demands for Ni worldwide, increasing Ni recycling and reducing waste are tangible goals for making substantial strides toward sustainability [24].

Recently, some nanoscale metal and metal oxide (M/MO_x_) heterostructures have been fabricated and exhibit high HER catalytic activity and stability; this is probably because of the synergistic effects of M and MO_x_, including Ni/NiO heterostructures [25–33]. However, these porous Ni/NiO composites are commonly powder and are fabricated through a complicated process, involving a sequence of hydrothermal, chemical reduction, and high-temperature processing [25, 26]. In addition, the preparation process of a traditional catalytic electrode that normally contains the addition of binder additives and carbon is also inconvenient. Hence, the search for economic, environmentally friendly, and feasible approaches to fabricate high-performance Ni-based catalysts for large-scale water splitting is still pursued and highly challenging.

In this work, we engineer the surface of commercial NF by quickly coating a cluster of Ni nanodots (NiNDs) (~ 2 nm) via electrochemical deposition. After drying in air, NiO/NiND composites can be obtained in a one-step procedure for constructing the binder-free and heteroatom-free NiO/NiNDs@NF electrodes. For comparison with commercial NF, the catalytic activity and durability of the NiO/NiNDs@NF electrodes toward HER and OER were greatly enhanced. The rough surface and porous structure of this composite with NiNDs can simultaneously expose more active sites with enhanced electrical conductivity. This NiO/NiND electrode consequently displays high activity and durability for electrocatalytic water splitting. The bifunctional catalytic electrode can enable highly efficient alkaline water electrolysis with 10 mA cm^−2^ at a cell voltage of only 1.70 V. More practically, a large-sized (*S* ~ 70 cm^2^) NiO/NiNDs@NF electrode fabricated using this method has also been demonstrated. This large-sized electrocatalytic electrode can enable alkaline water electrolysis with 13 mA cm^−2^ at 4.68 V (including electrical resistive loss in the electrolyte and electrode surfaces) [13] with superior durability. Importantly, these NiO/NiNDs can be easily removed using diluted HNO_3_ aqueous solution for the recovery and recycling of Ni foam and hydrated Ni(NO_3_)_2_. This NiO/NiNDs@NF electrode was prepared via one-step electrodeposition, which shortens the preparation process of the traditional electrode that normally contains added carbon and binder additives. With the low-cost, facile, and prompt fabrication strategy and the easy recycling property, this could be promising for water electrolysis devices used for large-scale H_2_ production.

## Experimental

### Fabrication of Electrocatalytic Electrodes

Commercial Ni foam (thickness: 0.5 mm) was first cleaned with acetone and then soaked in 0.5 M HNO_3_ for 10 min to remove the NiO from the surface. It was then washed with water and dried at room temperature. Ni nanoparticles were electrodeposited on the Ni foam in a N_2_-saturated acetonitrile solution containing 0.1 M Ni(NO_3_)_2_·6H_2_O (Residual water should first be eliminated through electrolysis.) ITO glass was used to electrodeposit and collect sample for N_2_ adsorption–desorption isothermal measurements. The electrochemical deposition process was conducted in a three-electrode system at a potential of − 1.46 V (vs. RHE) using an electrochemical workstation (GAMRY-111000) with Ag/Ag^+^ as a reference electrode, Pt wire as a counter electrode, and Ni foam as the working electrode. The mass loading of active materials can be adjusted by controlling the deposition time. The NiNDs@NF electrode was then washed with acetonitrile and dried in air at room temperature for 10 min. The NiO/NiNDs@NF electrode was prepared via oxidization of the pre-electrodeposited NiNDs in the air after drying. The prepared electrode can be directly used to collect the polarization curves or stored under vacuum for future use. The 120-s deposited electrode (loading of NiO/NiNDs was determined from the difference of the weight of NF before and after electrodeposition and was found to be ~ 1 mg cm^−2^) was used for the material characterizations and electrochemical tests. The large-sized NiO/NiNDs@NF electrode was fabricated using the same procedure with a large-sized carbon plate as the counter electrode. To prepare the Pt/C@NF electrodes, 1 mg of 20 wt% Pt/C or RuO_2_ (99.9%) was mixed with 90 μL of water, 50 μL of ethanol, and 10 μL of 5 wt% Nafion solution. The mixture was sonicated for 1 h to form a homogeneous ink. Then, 25 μL of the suspension was drop dried onto NF (0.5 cm^2^ loading of 1 mg cm^−2^ for the active mass).

### Materials Characterizations

The morphologies and structures of the NiO/NiND composites were investigated using scanning electron microscopy (SEM, LEO 1530) with an energy-dispersive X-ray (EDX) attachment (Zeiss) and using high-resolution transmission electron microscopy (HRTEM, JEOL JEM-2100). X-ray photoelectron spectroscopy (XPS, Thermo Scientific, Escalab 250Xi) was employed to analyze the composition of the NiO/NiND composites. N_2_ adsorption/desorption isotherms were obtained using a Quantachrome Autosorb-1 system at 77 K.

### Electrochemical Measurements

Cyclic voltammetry (CV) for HER and OER catalytic activity measurements was performed using a standard three-electrode system controlled by a GAMRY 11100 electrochemistry workstation. The current densities were calculated based on the projected geometric area of an electrode. All of the electrolytes were saturated by N_2_ bubbles for 30 min before the experiments. Different catalyst electrodes were used as the working electrode, a graphite plate was used as the counter electrode, and Ag/AgCl was used as the reference electrode. The reference was calibrated against and converted to the reversible hydrogen electrode (RHE; *E* (RHE) = *E* (Ag/AgCl) + 1.024 V; pH = 14). Water electrolysis measurements were carried out in a standard two-electrode system using the same deposited catalyst electrodes as the cathode and anode. Linear sweep voltammetry was carried out at 2 mV s^−1^ for the polarization curves. Chronopotentiometry was measured under a constant current density of 13 mA cm^−2^. All of the polarization curves in the three-electrode system were *iR*-corrected. Before measurements were made, a resistance test was conducted and *iR* compensation was applied using the GAMRY software. *R* is the equivalent series resistance, which was determined from electrochemical impedance spectroscopy (EIS). All of the data for the two-electrode electrolyzer were recorded without *iR* compensation. The faradaic efficiency was calculated by comparing the amount of gas determined from theoretical calculations and that determined from experimental measurements. H_2_ and O_2_ were collected using a water-drainage method, and the amounts of each were calculated using the moles of H_2_ and O_2_ generated from the overall water splitting with the ideal gas law. The theoretical amounts of H_2_ and O_2_ were calculated using *I*–*t* curve and by applying Faraday’s law [17]. The content of active materials was obtained by comparing the weight of the electrode before and after electrodeposition. EIS measurements were carried out by applying an AC voltage with 5 mV amplitude in a frequency range from 0.01 to 100 kHz at an overpotential of 0 V in 1.0 M KOH.

## Results and Discussion

### Fabrication of NiO/NiNDs Composites on NF

**Scheme 1:**
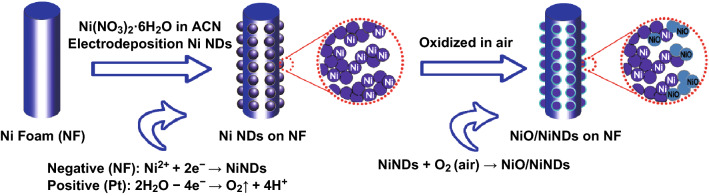
Schematic illustration of the fabrication process of NiO/NiND composites on nickel foam and the corresponding reaction during and after electrodeposition

The fabrication process of NiO/NiND composites is illustrated in Scheme 1. First, NiND clusters were deposited on NF via an electrochemical reduction process of Ni^2+^ → Ni^0^ in N_2_-saturated acetonitrile (ACN) solution (Fig. S1 and Movie S1). The corresponding reactions are also shown in Scheme 1, in which Ni^2+^ was reduced to Ni^0^ on the negative electrode instead of the H_2_ evolution reaction occurring in aqueous solution at the same potential (Fig. S2). This is because of the wider potential window of ACN (Fig. S3). Moreover, the black film composed of NiNDs was accessible only in ACN instead of other common organic solvents (e.g., DMSO, THF, DMF, and ethanol) that have a similar wide potential window. This is probably because of the higher thermodynamic stability of [Ni(ACN)_6_]^2+^ [34]. It has also been reported that the higher donor property of ACN prevents the directional growth of nucleation for the formation of a compact metallic film. The same effect of ACN has also been observed during electrodeposition of Co or Fe nanoparticles using the ACN solution [35, 36]. Meanwhile, these deposited nanoparticles all showed high reducibility (Fig. S4), providing a convenient and quick method for fabricating nanoscale NiO/Ni composites. Consequently, the NiO/NiNDs@NF electrode can be obtained instantly when these electrodeposited electrodes were exposed to air. This strategy, which does not include adding carbon and binder additives, greatly shortens the preparation process of the traditional electrode.

### Materials Characterization

**Fig. 1:**
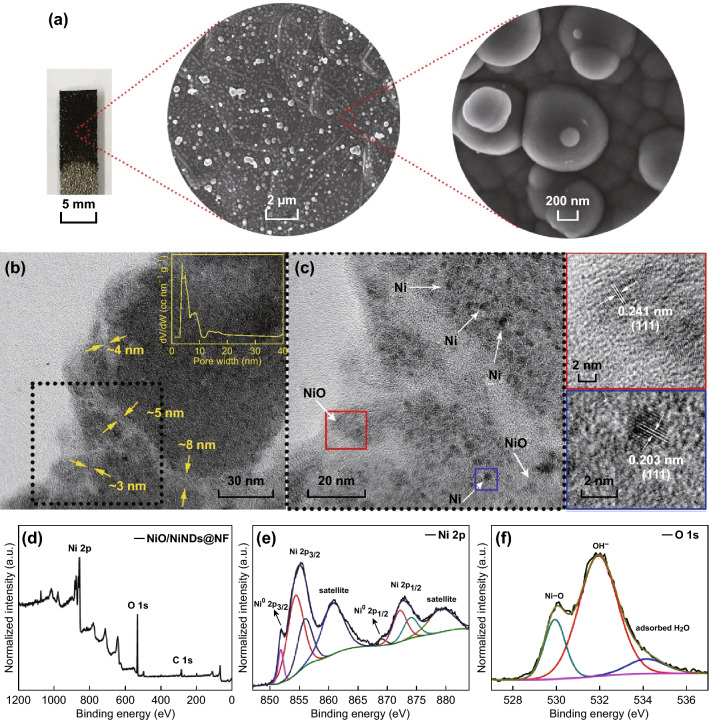
Structure and components of NiO/NiND composites after 120 s of deposition. **a** Photographic images of the NiO/NiNDs@NF electrode, SEM image, and the corresponding magnified SEM image. **b** TEM and **c** HRTEM images (the magnified view of the NiO and Ni crystal lattice is in the red and blue frame respectively); inset of b is the corresponding BJH pore size distribution curve shown by the yellow line; **d**–**f** full XPS spectrum, and XPS spectra of Ni 2*p* and O 1*s*. (Color figure online)

The morphologies and structures of the NiO/NiND composites were first examined using SEM and TEM. A representative SEM image of the NiO/NiND composites is shown in Fig. 1a. Integrated spherical nodules formed by NiND clusters with different sizes were observed on the NF. The whole deposition process is described in Figs. S5, S6. With an increase in the deposition time, the thickness of the film increased gradually and finally became more easily detached from NF (Fig. S5). Therefore, the 120-s deposited materials were used for characterization and fabrication of the electrocatalytic electrodes. The growth process of NiND clusters can be observed from the low-scale SEM images (Fig. S6). At the beginning of the deposition process, plenty of NiND clusters formed uniformly on the surface of NF, and then, these clusters grew, accumulated, and integrated with each other. The homogeneous spatial distributions of Ni and O in the composites are clearly verified by the corresponding energy-dispersive spectroscopy (EDS) mapping images, and the atomic ratio of Ni/O approached 3:1 (Fig. S7). The structure of the NiO/NiND composites was further investigated using high-resolution transmission electron microscopy (HRTEM) measurements, which are shown in Fig. 1b, c. Figure 1b shows the porous structures among the nanoparticles, and it is observed that the hollow space is between 3 ~ 8 nm. The hollow space is beneficial for the penetration and diffusion of electrolytes. Meanwhile, the specific surface area and pore size distribution of the NiO/NiND composites were examined using N_2_ adsorption–desorption isothermal measurements. The NiO/NiND sample shows an apparent hysteresis loop in the sorption isotherm, indicating its mesoporous structure (Fig. S8). The pore size distribution curve was obtained using the Barrett–Joyner–Halenda (BJH) method, and the results show a narrow range of mesopores between 3 ~ 10 nm and a broad peak centered at 15 nm (inset of Fig. 1b). From calculations, the Brunauer–Emmett–Teller (BET) surface area of the NiO/NiND composite is 69 m^2^ g^−1^. HRTEM images in Fig. 1c further demonstrate that the spherical nodules are composed of nanoparticles that are 2 nm in size. The interplanar spacing of 0.203 nm matches well with the *d*_111_ spacing of metal Ni (JCPDS No. 65-2865), and another lattice fringe spacing of 0.241 nm corresponds to the (111) plane of NiO (JCPDS No. 47-1049). These observations indicate that the obtained composites consist of both metallic NiO and Ni nanoparticles, providing a favorable low resistance pathway for electron transfer. To further confirm the NiO/NiND composites, XPS analyses were conducted. Figure 1d displays the full spectrum of the NiO/NiND composite, in which the atomic ratio of Ni/O is approximately 3:1. The high-resolution XPS spectra in the Ni 2*p* and O 1*s* regions are presented in Fig. 1e, f. The peaks centered at 854.4 and 856.2 eV correspond to Ni (II) 2*p*_3/2_, and peaks at 872.1 and 873.9 eV originate from Ni (II) 2*p*_1/2_ [37, 38]. The characteristic peaks of Ni^0^ are located at 852.4 and 869.6 eV, respectively, for Ni 2*p*_3/2_ and Ni 2*p*_1/2_ [39]. Satellite peaks of Ni 2*p*_3/2_ and 2*p*_1/2_ are observed at 860.7 and 879.3 eV. In addition to the peaks of Ni^2+^, the noticeable characteristic peaks of Ni^0^ are also observed, indicating the existence of metal Ni. The O 1*s* signal shows three peaks located at 530, 532, and 534 eV, which can be assigned to O^2−^ in NiO, hydroxyl groups (OH^−^), and surface-adsorbed H_2_O, respectively. These observations are in accord with the results of reported NiO/Ni composites [26, 27]. Moreover, a lot of carbon-containing functional groups are observed in the high-resolution XPS spectrum of C 1*s* (Fig. S9). These groups should come from coordinating ACN molecules or their derivatives because of the high thermodynamic stability of [Ni(ACN)_6_]^2+^. During the reduction process of Ni^2+^ to Ni in the electrodeposition of NiNDs, some of the ACN molecules or derived groups are retained, and this probably leads to the formation of NiNDs. Similar results have been reported in the previous work for the electrodeposition of cobalt and iron nanoparticles using the ACN solution [35, 36].

### Electrochemical Properties of NiO/NiNDs@NF

**Fig. 2:**
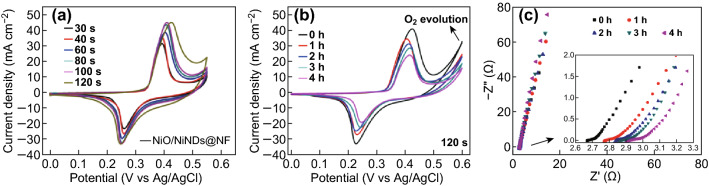
CV characteristics (scan rate of 5 mV s^−1^) of **a** NiO/NiNDs@NF electrodes with different deposition time and **b** 120-s deposited NiO/NiNDs@NF electrodes before and after heating at 200 °C. **c** Corresponding Nyquist plots of the curves shown in panel b over the frequency range of 0.01 Hz–100 kHz with 5 mV AC amplitude at an overpotential of 0 V in 1.0 M KOH. Inset is a local enlargement of the high-frequency region

To investigate the electrochemical kinetics of the NiO/NiNDs@NF electrode, a three-electrode system with 1 M KOH as an aqueous electrolyte was used to measure the electrochemical performances of the NiO/NiNDs@NF electrodes. The CV curves of the NiO/NiNDs@NF electrode with different deposition times are displayed in Fig. 2a. Each CV curve has the same pair of distinct redox peaks in the potential range of 0–0.55 V at a scan rate of 5 mV s^−1^, indicating the faradaic redox reaction [40]:

$${\text{NiO}} + {\text{OH}}^{ - } \rightleftharpoons {\text{NiOOH}} + {\text{e}}^{ - 1}$$

The corresponding right shift of the anodic potentials may result from the increased conductivity that occurs with an increase in the internal metallic Ni content of the NiO/NiND composite, which results in decreased polarization loss [41, 42]. By re-plotting peak current *versus* scan rate, both the anodic and cathodic plots show a linear relationship regardless of scan rate (Fig. S10), suggesting a controlled semi-infinite diffusion process during cycling. To investigate the effects of NiNDs on the electrochemical performance of NiO/NiND composites, the NiO/NiNDs@NF electrodes were heated at 200 °C in air to aggravate the oxidation of NiNDs. Figure 2b shows the CV curves of the NiO/NiNDs@NF electrodes with different heating times. The redox peaks for the NiO/NiNDs@NF electrode without heating treatment show a larger peak area and higher anodic potential. With an increase in the heating time, the stable redox peaks accordingly become smaller, and meanwhile the catalytic current for OER also gradually weakens. This may result from the decreased conductivity that occurs because of the decrease in metal Ni content. The corresponding EIS spectra of the treated NiO/NiNDs@NF electrodes are shown in Fig. 2c. The near-vertical slopes of the Nyquist plots in the low-frequency region reveal that the electrodes display a capacitive-like behavior. This behavior indicates the fast diffusion of the electrolyte (inset of Fig. 2c) [43]. The charge transfer resistance (*R*_ct_) obtained from the simulated diameter of the resistor–capacitor loop increases with the decrease in Ni content, which is in accord with the increase of *R*_s_ and decrease in electrical conductivity for the NiO/NiND composites (Fig. S11). These results further reveal that the metallic NiNDs are responsible for the high conductivity of the NiO/NiND composites, which endows the NiO/NiNDs@NF electrode with a higher electrochemical performance to enhance its catalytic properties.

### Catalytic Activity of NiO/NiNDs@NF

**Fig. 3:**
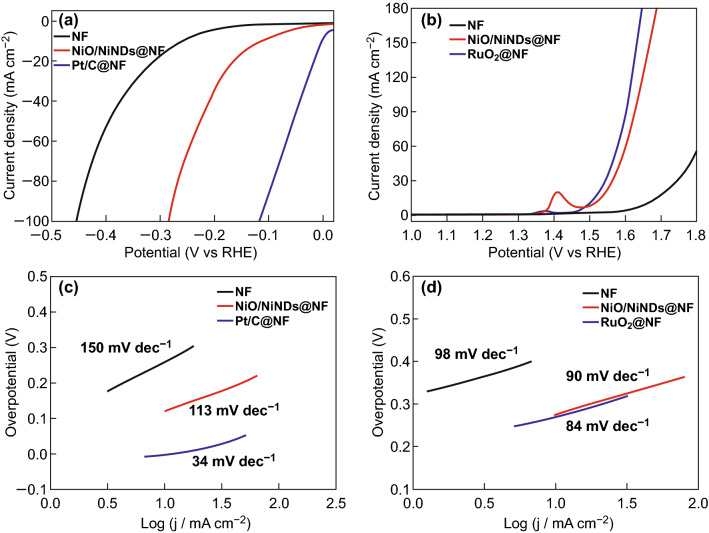
**a** HER and **b** OER characteristics of different catalyst electrodes under 1 mg cm^−2^ loading determined using linear sweep voltammetry with a three-electrode configuration in 1 M KOH aqueous electrolyte. All of the scan rates were 2 mV s^−1^. Tafel plots of different catalyst electrodes for **c** HER and **d** OER

Catalytic activity of electrodes toward HER and OER was evaluated using linear sweep voltammetry (LSV) with a three-electrode system in 1.0 M KOH and with a scan rate of 2 mV s^−1^. For comparison, the catalytic activity toward HER of bare NF, the commercial 20 wt% Pt/C, and RuO_2_ deposited on NF with the same loading (weight density of 1 mg cm^−2^) were also tested. As-measured reaction currents do not directly reflect the intrinsic behavior of catalysts because of the effect of ohmic resistance, and thus, an *iR* correction was applied to all of the initial data for further analysis. As seen in Fig. 3a, the electrocatalytic performance of NiO/NiNDs@NF is much better than that of NF. To achieve a current density of 10 mA cm^−2^, the NiO/NiNDs@NF electrode requires an overpotential (*η*_HER_) of 119 mV, which is 119 mV higher than that of the Pt/C@NF electrode (0 mV). This overpotential is smaller than the behavior of other reported Ni- and Co-based HER electrocatalysts when operated in alkaline aqueous solution. Such catalysts include the Ni inverse opal (240 mV) [44], Ni_2_P nanoparticles (180 mV) [45], Ni_3_S_2_/carbon nanotube composites (350 mV) [46], Ni_x_Co_10 − x_/C nanoflakes (370 mV) [47], and 3DOM/m Ni (171 mV) [48]. Meanwhile, the overpotential (164 mV) for 20 mA cm^−2^ is also smaller or comparable to that of other modified NF (Table S1) and reported Ni- and Co-based bifunctional catalysts, including Ni_2_P (255 mV) [49], NiCo_2_S_4_/carbon cloth (190 mV) [50], and Ni_5_P_4_/Ni foil (169 mV) [51]. Figure 3c shows the Tafel plots of the bare NF, NiO/NiNDs@NF, and Pt/C@NF. The Tafel slopes of 150, 113, and 34 mV dec^−1^ for NF, NiO/NiNDs@NF, and Pt/C@NF, respectively, were determined from the fitting of the linear region of the corresponding Tafel plots using the Tafel equation (*η* = *b* log*j* + *a*, where *j* is the current density and *b* is the Tafel slope). The relatively small Tafel slope of NiO/NiNDs@NF indicates a faster increase in the HER rate with an increase in potential.

The OER characteristics of these different electrodes are shown in Fig. 3b. The NiO/NiNDs@NF electrode also exhibits excellent catalytic activity toward OER. In the three-electrode configuration, the NiO/NiNDs@NF electrode has a much higher current density than the bare NF and an onset potential as low as 1.55 V versus RHE. It requires an overpotential (*η*_OER_) of 360 mV to reach a (projected geometric area) current density of 50 mA cm^−2^, which is 21 mV more than that of the RuO_2_@NF electrode. Meanwhile, this overpotential is comparable to the behavior of other modified NF (Table S1) and reported state-of-the-art Ni- and Co-based bifunctional catalysts, such as Ni–P/Cu foam (410 mV) [52], Ni_3_Se_2_/Cu foam (343 mV) [53], Ni_5_P_4_/Ni foil (363 mV) [51], and CoSe/Ti mesh (341 mV) [54]. The anodic peak at 1.41 V corresponds to the oxidation of NiO. Moreover, a minor anodic peak is observed at 1.35 V in the magnified LSV curve, which corresponds to the oxidation of NiNDs (Fig. S12). The Tafel slope for the NiO/NiNDs@NF electrode is calculated to be 90 mV dec^−1^, which is close to the corresponding value of the RuO_2_@NF (84 mV dec^−1^) electrode and smaller than the corresponding value of bare NF (98 mV dec^−1^) (Fig. 3d). Moreover, to further investigate the effects of NiNDs, we used LSV and the heat-treated NiO/NiNDs@NF as the electrode to test its electrocatalytic properties for HER and OER. Obviously, the heating treatment (at 200 °C in air) has large effects on HER and OER performance, which gradually declined with an increase in the heating time (Fig. S13). These results further indicate that the NiNDs play an important role in the excellent electrocatalytic activity toward HER and OER.

**Fig. 4:**
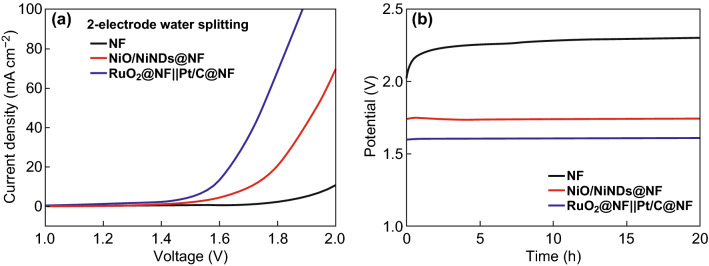
**a** Overall water-splitting characteristics of different catalyst electrodes in a two-electrode configuration. **b** Chronopotentiometric curves of water electrolysis for different catalyst electrodes in a two-electrode configuration with a constant current density of 13 mA cm^−2^ (without *iR* correction)

To test the applicability as a bifunctional electrocatalyst for overall water electrolysis, an electrolyzer that used a NiO/NiNDs@NF electrode in 1.0 M KOH as both the anode and cathode was assembled (NiO/NiNDs@NF‖NiO/NiNDs@NF). As a control, another water electrolyzer NF‖NF and RuO_2_@NF‖Pt/C@NF are also made. Figure 4a shows the polarization curves of the different electrolyzers. Overall, NiO/NiNDs@NF‖NiO/NiNDs@NF can achieve 10 mA cm^−2^ water-splitting current by applying just 1.7 V across the electrodes, which is a much better performance than that of bare NF. This potential is only 124 mV more than that of RuO_2_@NF‖Pt/C@NF. Although this value is larger than that of RuO_2_@NF‖Pt/C@NF, it is comparable to the values required by electrolyzers that are based on the other reported state-of-the-art Ni- and Co-based bifunctional catalysts, such as Ni_3_S_2_/Ni foam‖Ni_3_S_2_/Ni foam (above 1.7 V) [55], NiFe LDH/Ni foam‖NiFe LDH/Ni foam (1.7 V) [56], Ni_5_P_4_/Ni foil‖Ni_5_P_4_/Ni foil (1.68 V) [51], NiCo_2_S_4_/carbon cloth‖NiCo_2_S_4_/carbon cloth (1.68 V) [50], and NiS/Ni foam‖NiS/Ni foam (above 1.64 V) [57]. All of the above results demonstrate the exciting potential of this Ni-based electrode for water electrolysis.

### Catalytic Stability of NiO/NiNDs@NF

Long-term electrocatalytic stability is another important criterion for water-splitting electrocatalysts because a longer lifetime of a device reduces the cost of the resulting H_2_. To assess the durability of the NiO/NiNDs@NF electrodes, an applied current density was set at 13 mA cm^−2^. A constant potential of 1.74 V can be well maintained for at least 20 h without any decay (Fig. 4b), and this indicates the high stability of the NiO/NiNDs@NF electrode. For comparison, the applied potential of RuO_2_@NF‖Pt/C@NF (1.61 V) is nearly 120 mV less than that of the NiO/NiNDs@NF electrode, and the NF (2.0 V) electrodes show a poor electrocatalytic activity and stability. The potential of the NF electrode increased greatly during the first few hours, suggesting a dramatic decline in the catalytic activity. Meanwhile, the faradaic efficiency during the overall water splitting is almost 100% for both HER and OER, and the molar ratio of H_2_ to O_2_ remains at 2:1 (Fig. S14). After the durability assessments, the NiO/NiNDs@NF electrodes were also tested using SEM and TEM, and the results indicate no topographic changes. This highlights the superior structural robustness of the NiO/NiND composites during the electrocatalytic HER and OER processes (Fig. S15). However, the NiNDs content decreased obviously after the long-term OER durability test (Fig. S16). The survival of some of the NiNDs is probably because of the existence of carbon-containing groups on the surface of NiNDs, which may retard the oxidation of NiNDs. It is worth mentioning that the excellent catalytic stability of NiO/NiND composites should be attributed to the NiND survival in the electrochemical tests (Fig. S17).

### Proposed Mechanism of Electrocatalytic Property of NiO/Ni composites

On the basis of these results, we propose three explanations for the superior electrocatalytic performance of NiO/Ni composites: First, the presence of metallic Ni nanoparticles increases the conductivity of the catalyst; this is beneficial for electron transport through NiO and improves the catalytic stability. Second, the rough surface and porous nanostructure enhance electron transfer by increasing the reaction area and preventing bubbles from growing; in turn, this increases the rate of electrolysis. Third, the synergistic effect of surface NiO and Ni nanoparticles can further improve the catalytic activity of NiO/Ni composites. On the one hand, Ni supplies the active catalytic sites and highly improves the conductivity of the catalyst. On the other hand, the OH^−^ that is generated by H_2_O splitting can preferentially attach to a NiO site at the interface because of strong electrostatic affinity to the locally positively charged Ni^2+^ species and the larger number of unfilled *d* orbitals in Ni^2+^ than in Ni metal [25].

### Fabrication of Large-sized NiO/NiNDs@NF Electrode

**Fig. 5:**
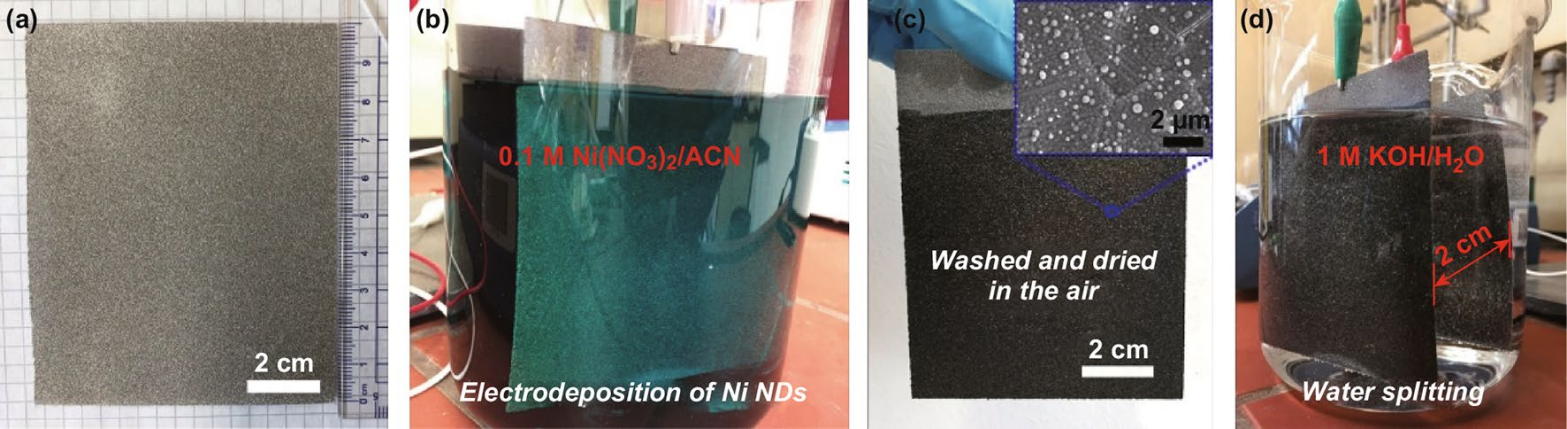
Photographs of the preparation and application process of large-area NiO/NiNDs@NF electrodes: **a** Ni foam that is 80 cm^2^. **b** Electrodeposition process of NiNDs on NF. **c** The obtained NiO/NiNDs@NF electrode that has an effective geometric surface area of 70 cm^2^ (inset is an SEM image). **d** Generation of H_2_ and O_2_ bubbles on the NiO/NiNDs@NF electrode with a constant current density of 13 mA cm^−2^ (see SI for a video)

For further practical application, overall water splitting was also investigated in a large-sized catalytic electrode. As described in Fig. 5, NF that was 80 cm^2^ was used to fabricate the NiO/NiNDs@NF electrode via this method (Fig. 5a). Electrodeposition of NiNDs was conducted in the same way as described above, using a large-sized carbon plate as the counter electrode (Fig. 5b). During the electrodeposition process, formation of a black and uniform film was observed on the surface of NF. After electrodepositing and drying in air, a large-sized NiO/NiNDs@NF electrode with a 70 cm^2^ active area was obtained (Fig. 5c). The same surface topography (of integrated spherical nodules as small-sized electrodes) described above is seen in the SEM images (inset of Fig. 5c). As expected, the large-sized NiO/NiNDs@NF electrodes were directly used to electrocatalyze water decomposition in 1 M KOH (Fig. 5d and Movie S2). All of these results demonstrate the feasibility of this method for large-scale production and practical applications in a water electrolyzer. In contrast to the previous small-sized electrodes (*S*_electrode_ ~ 0.5 cm^2^), this large-sized NiO/NiNDs@NF electrodes enable an alkaline water electrolyzer with 13 mA cm^−2^ at 4.68 V (without *iR* correction). This illustrates the high applied potential for large-scale water electrolysis. The high potential drop mainly results from the larger additional resistance (including resistance losses in the electrolyte, substrate, and contact interface) [13]. More detailed work to decrease the resistance loss is under way.

**Fig. 6:**
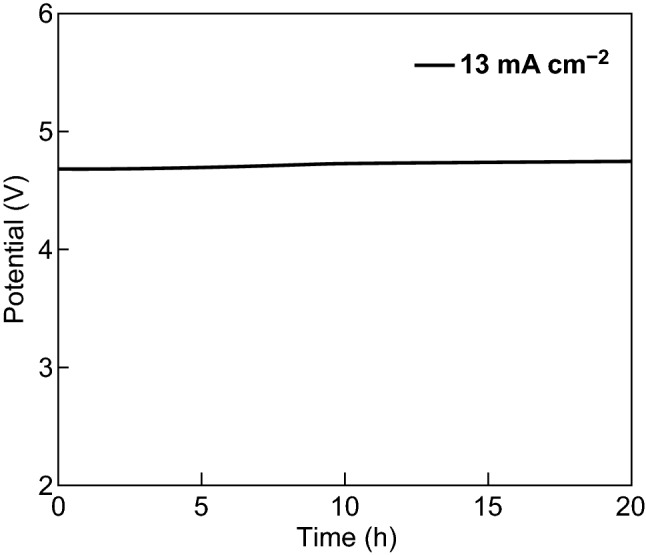
Chronopotentiometric curve of water electrolysis using large-sized NiO/NiNDs@NF‖NiO/NiNDs@NF (*S* ~ 70 cm^2^) with a constant current density of 13 mA cm^−2^

The large-sized water electrolyzer also exhibited superior durability (Fig. 6). The excellent stability and fast preparation process greatly simplified the manufacturing procedures and saved time, which is advantageous for practical applications.

### Recycling NF and Ni(NO_3_)_2_

These NiO/NiND composites can quickly dissolve in acid solution to form the corresponding salt of Ni^2+^, leading to the regeneration of NF. As seen in Fig. S18a, the color of the NiO/NiNDs@NF electrode changes gradually from black to silver-white when it is immersed in 0.5 M HNO_3_ solution. The SEM images further indicate that the spherical nodules on the surface of NF can be completely dissolved in 0.5 M HNO_3_ solution for 20 min (Fig. S18b). In addition, the corrosion behavior of pure NF in 0.5 M HNO_3_ solution has been investigated. No vigorous reaction was observed, and there was a merely 4% decrease in the weight of NF after 20 min of the immersion treatment (Fig. S18c). This dissolution process of NF in NiO/NiNDs@NF should be slow because of the surface coating of NiO/NiNDs. Meanwhile, the dissolved product of NF and the concentrated treatment solution can also be used as a source of Ni(NO_3_)_2_. Thus, the regenerated Ni foam can be recycled and used in the fabrication of the NiO/NiNDs@NF electrodes. In total, the whole recovery process of Ni foam and Ni(NO_3_)_2_ is simple and environmentally friendly without any emission of toxic gases or wasted energy. All of the above results demonstrate the convenient fabrication and recyclability of the electrodes for practical applications in water electrolysis.

## Conclusion

To construct high-performance, low-cost, and environmentally friendly Ni-based catalytic electrodes for water-splitting, binder-free, heteroatom-free, and recyclable NiO/NiNDs@NF bifunctional catalytic electrodes were fabricated using a one-step quick electrodeposition method. Typically, active Ni nanodot clusters were electrodeposited on Ni foam in acetonitrile solution. After drying in the air, the NiO/NiND composites were obtained, leading to binder-free and heteroatom-free NiO/NiNDs@NF catalytic electrodes, which have superior performance during HER and OER processes. A large-sized (*S* ~ 70 cm^2^) catalytic electrode with high durability was also fabricated using this method. Importantly, the recovery process of the raw materials of these electrodes is convenient and environmentally friendly for their recycling use. This method provides a simple and fast technology for preparing recyclable Ni-based bifunctional electrocatalytic materials for large-scale real-world water-splitting electrolyzers.

## Electronic Supplementary Material

Below is the link to the electronic supplementary material.

Supplementary material 1 (AVI 2214 kb)

Supplementary material 2 (AVI 1047 kb)

Supplementary material 3 (PDF 1434 kb)
