# Supplementary material for: Prompt Electrodeposition of Ni Nanodots on Ni Foam to Construct a High-Performance Water-Splitting Electrode: Efficient, Scalable, and Recyclable
Source: Nanomicro Lett. 2019 May 16;11:41. doi: 10.1007/s40820-019-0269-x (PMC7770909; doi:10.1007/s40820-019-0269-x)
Supplement: Supplementary file 3 — Supplementary material 3 (PDF 1434 kb) [file 40820_2019_269_MOESM3_ESM.pdf]

Supporting Information for

Prompt Electrodeposition of Ni Nanodots on Ni Foam to Construct a High-Performance Water Splitting Electrode: Efficient, Scalable, and Recyclable

Hongtao Yu[1, 2], Ting Quan[1], Shilin Mei[1], Zdravko Kochovski[1], Wei Huang[2, *], Hong Meng[2], Yan Lu[1, 3, *]

[1]Soft Matter and Functional Materials, Helmholtz-Zentrum Berlin für Materialien und Energie GmbH, Hahn-Meitner Platz 1, 14109, Berlin, Germany.

[2]Key Lab for Flexible Electronics & Institute of Advanced Materials, Jiangsu National Synergistic Innovation Center for Advanced Materials (SICAM), Nanjing Tech University, 30 South Puzhu Road, Nanjing, People's Republic of China

[3]Institude of Chemistry, University of Potsdam, 14467, Potsdam, Germany

*Corresponding authors. E-mail: yan.lu@helmholtz-berlin.de (Yan Lu); iamwhuang@njtech.edu.cn (Wei Huang)

Supplementary Figures and Table

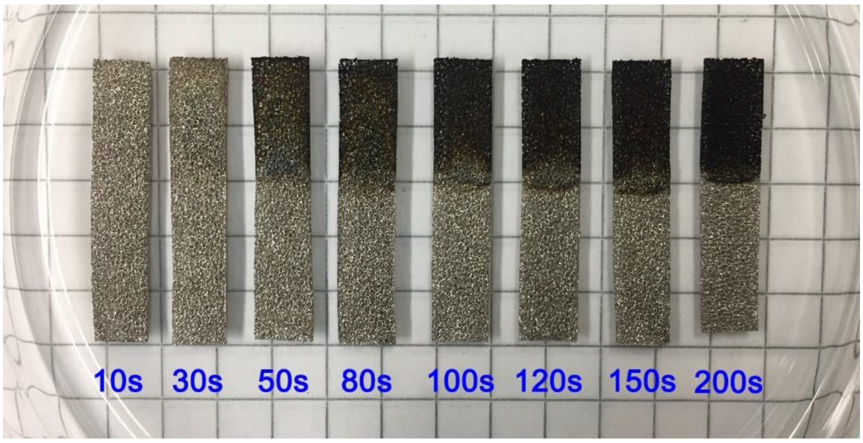

Fig. S1 Digital photograph of NiO/NiNDs@NF with different electrodeposition time. During this process, an apparent color change of NF was observed. The color gradually changed from silver-white to black because of the increase in deposited NiNDs

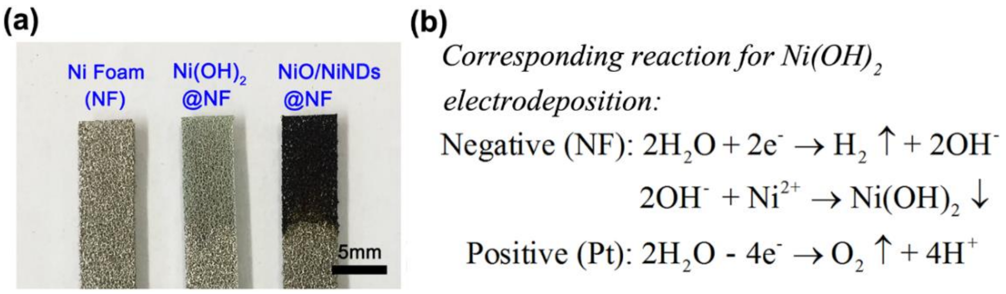

Fig. S2 a Digital photograph of Ni foam, $Ni(OH)_2$@NF, and NiO/NiNDs@NF. **b** Corresponding chemical reaction for $Ni(OH)_2$ electrodeposition in aqueous solution

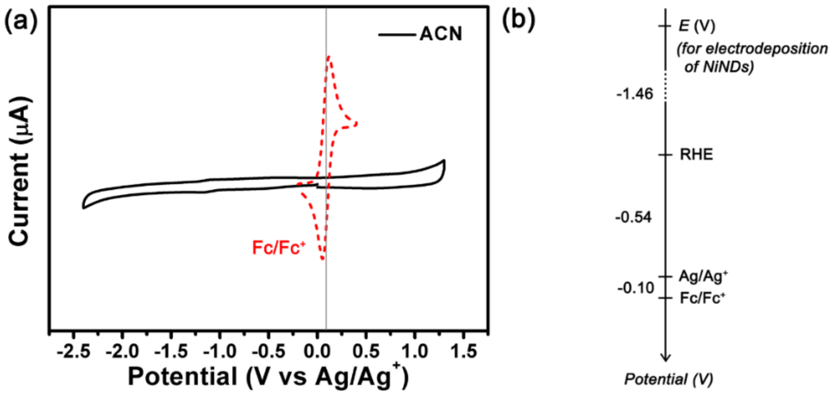

Fig. S3 a Potential window of ACN. CV measurement was performed using a standard three-electrode system controlled by a GAMRY 11100 electrochemistry workstation. GCE was used as the working electrode, Pt wire was used as the counter electrode and Ag/Ag+ was used as the reference electrode. The scan rate was 50 mV s^{-1}. **b** The reference for electrodeposition was calibrated against and converted to the reversible hydrogen electrode (RHE) through the potential of Fc/Fc^+ [S1]

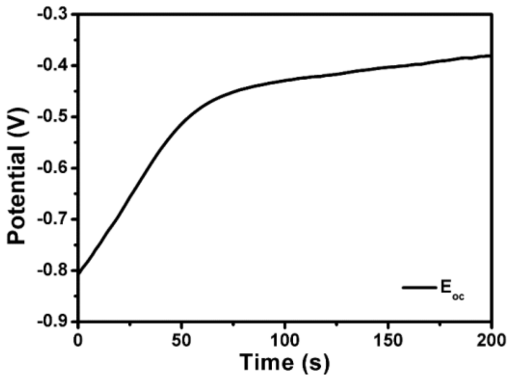

Fig. S4 Open circuit potential of a freshly obtained NiNDs@NF electrode, which shows a high negative open circuit potential (E_{oc}). This potential dropped drastically after the first dozens of seconds because of the quick oxidation of external NiNDs ($Ni^0 \rightarrow Ni^{2+}$). The decreasing in this value becomes gentle with further oxidation of the internal NiNDs

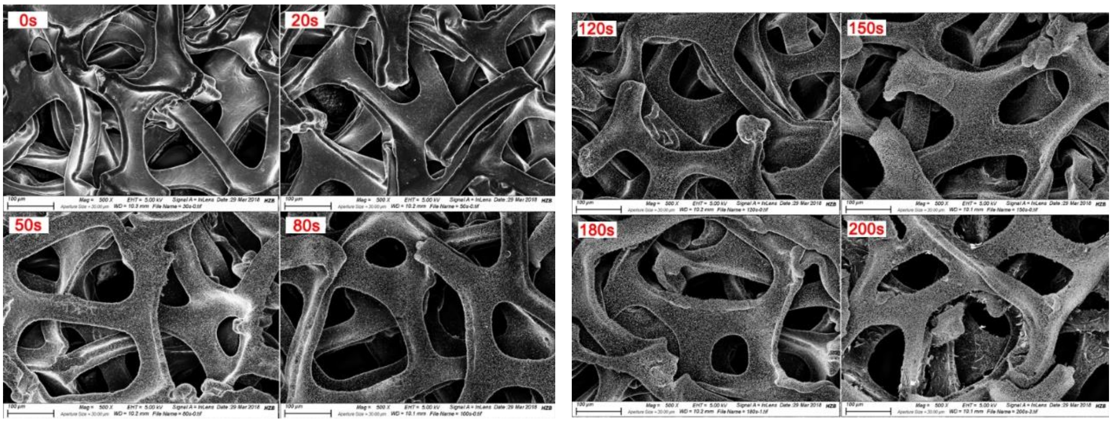

Fig. S5 Large-scale SEM images of NiO/NiNDs@NF with different electrodeposition time

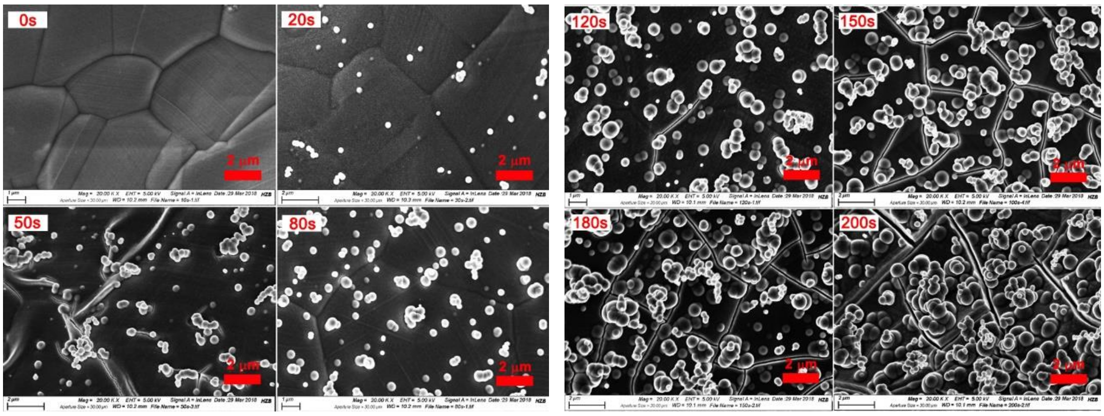

Fig. S6 Low-scale SEM images of NiO/NiNDs@NF with different electrodepositon time. Integrated spherical nodules of NiND clusters with different sizes formed gradually on NF

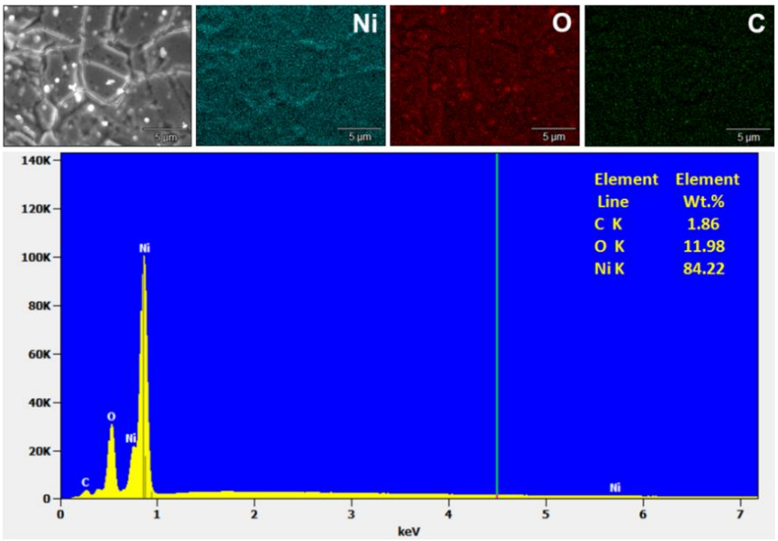

Fig. S7 EDX spectrum and mapping of Ni, O, and C element of the NiO/NiNDs@NF

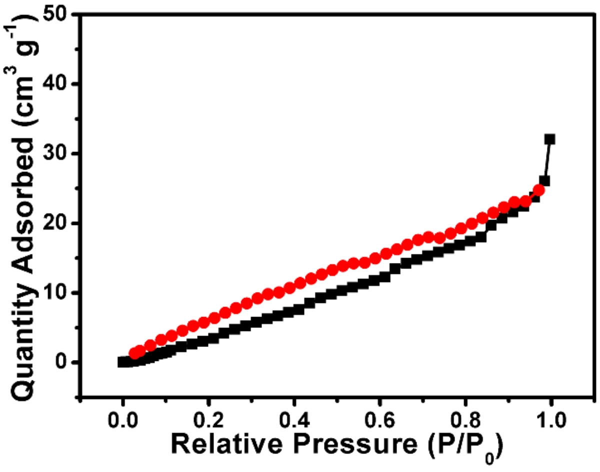

Fig. S8 Nitrogen sorption isotherms of the NiO/NiNDs samples

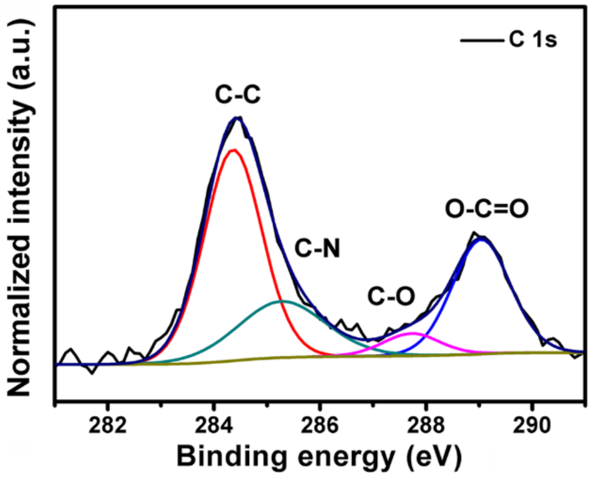

Fig. S9 High-resolution XPS C 1s spectra

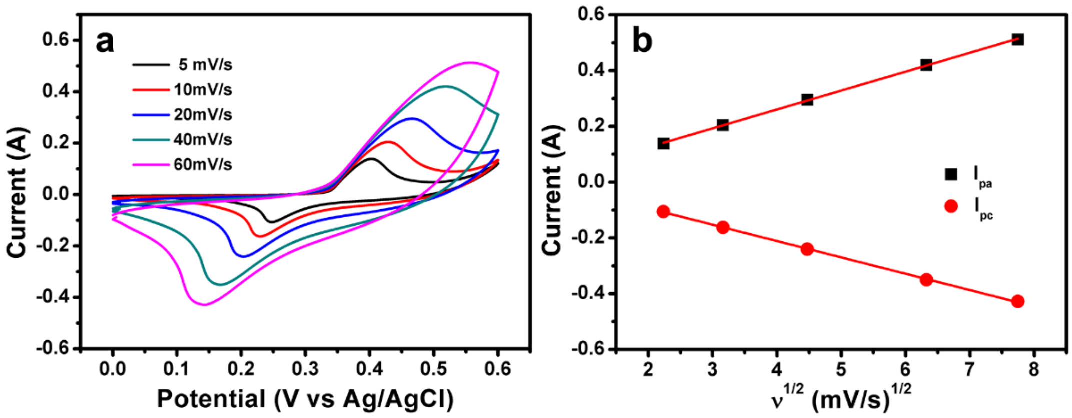

Fig. S10 a CV curves of the NiO/NiNDs@NF electrode with different scan rates. **b** Anodic and cathodic peak current densities obtained from the CV curves and plotted as a function of the square root of the scan rates

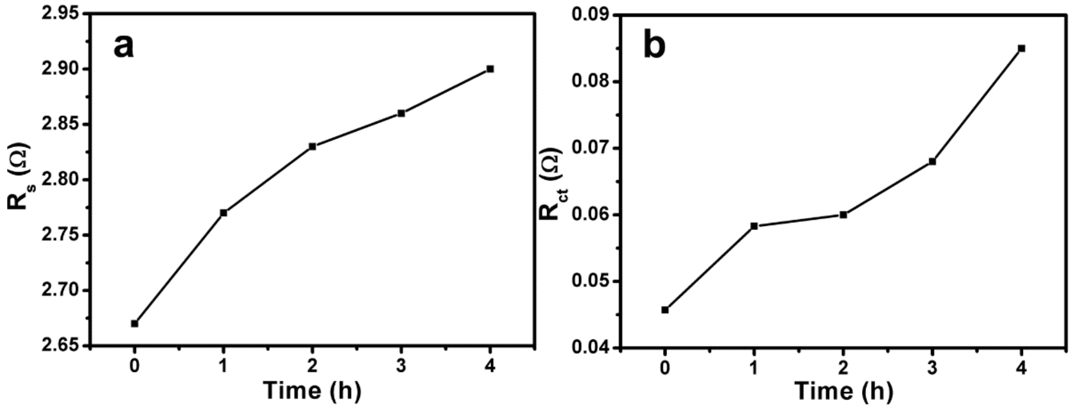

Fig. S11 a R_s and **b** R_{ct} of EIS spectra of NiO/NiNDs@NF electrodes as a function of the heating time in a 200 °C oven

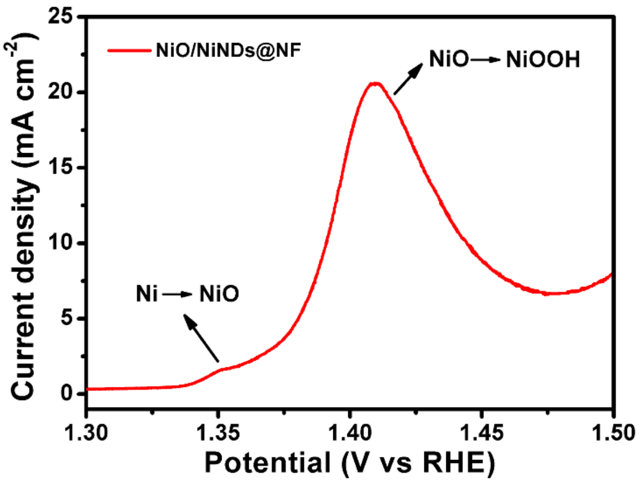

Fig. S12 Magnified LSV curve of NiO/NiNDs for OER

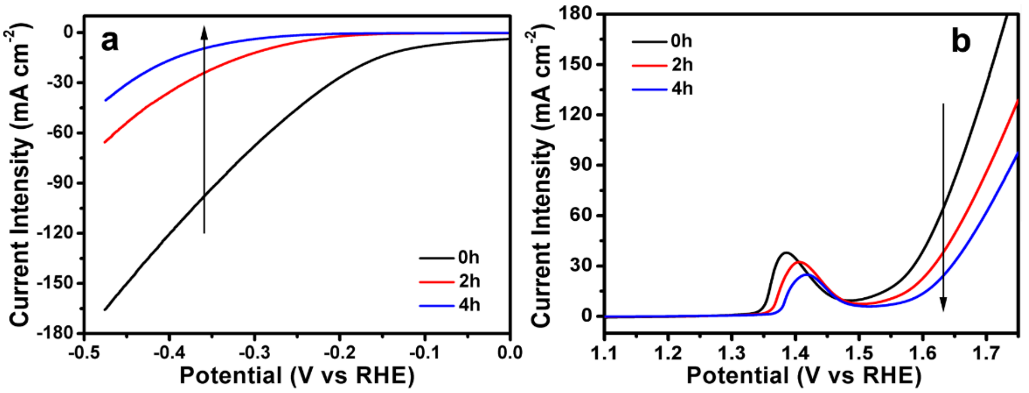

Fig. S13 Electrocatalytic performance of the 120 s' deposited NiO/NiNDs@NF electrodes with different heating time (0, 2, and 4 h) at 200 °C. Linear sweep voltametry of the corresponding NiO/NiNDs@NF electrodes in 1M KOH at a scan rate of 5 mV s^{-1} for **a** HER and **b** OER

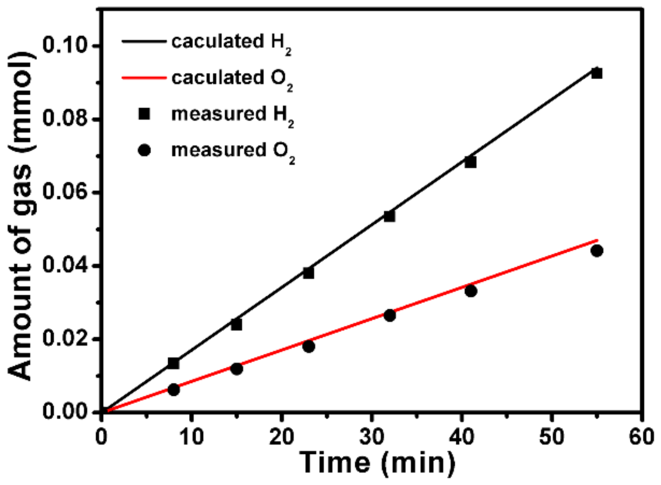

Fig. S14 Amount of gas theoretically calculated and experimentally measured versus time for NiO/NiNDs in 1.0 M KOH

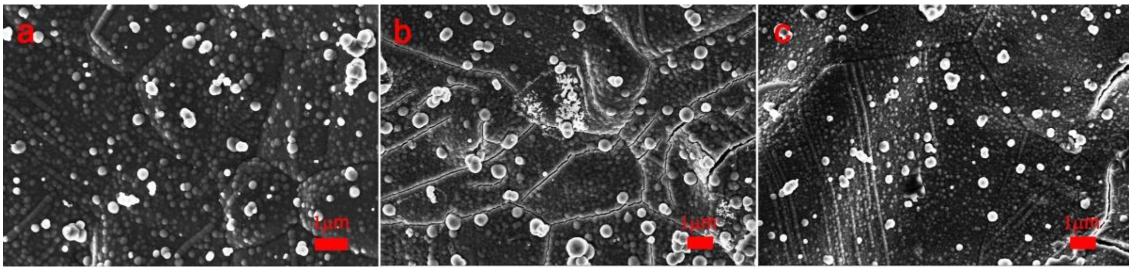

Fig. S15 Morphologies of NiO/NiNDs@NF **a** before and **b** after long-term HER and **c** OER tests

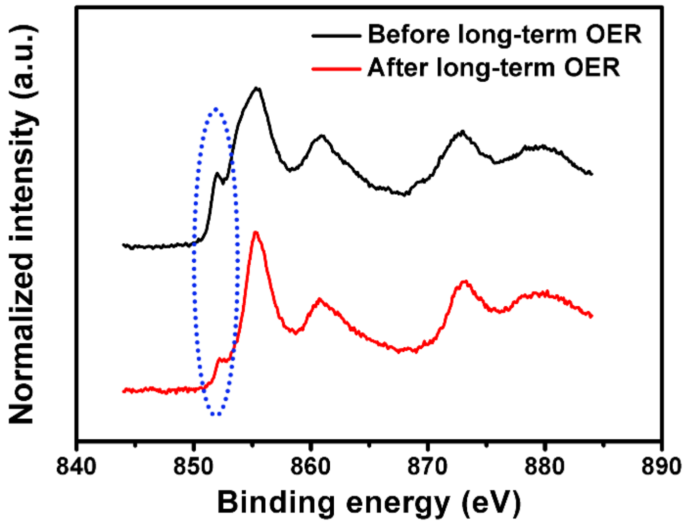

Fig. S16 High-resolution XPS Ni 2p spectra before and after long-term OER measurement

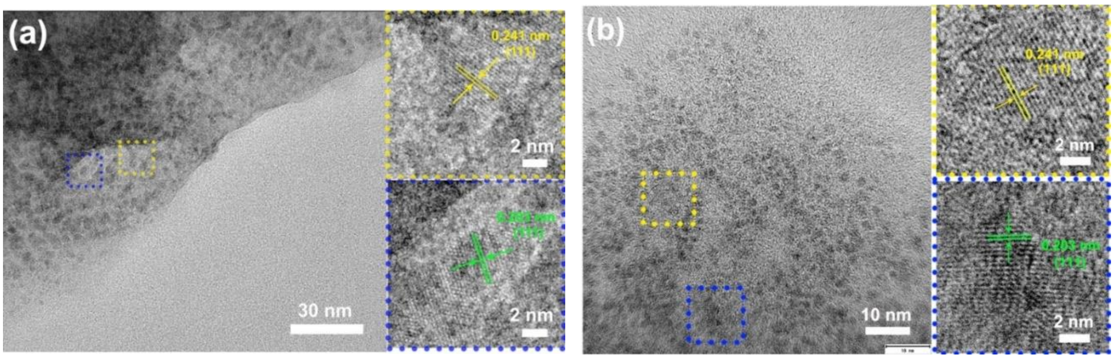

Fig. S17 HRTEM images of NiO/NiNDs@NF after long-term **a** HER and **b** OER tests. The lattice fringe spacing of 0.241 nm corresponds to the (111) plane of NiO (JCPDS (Joint Committee on Powder Diffraction Standards) No. 47-1049). Another interplanar spacing of 0.203 nm matches well with the d_{111} spacing of metal Ni (JCPDS No. 65-2865), revealing that the NiND in the NiO/NiND composites are stable during long-term HER and OER processes

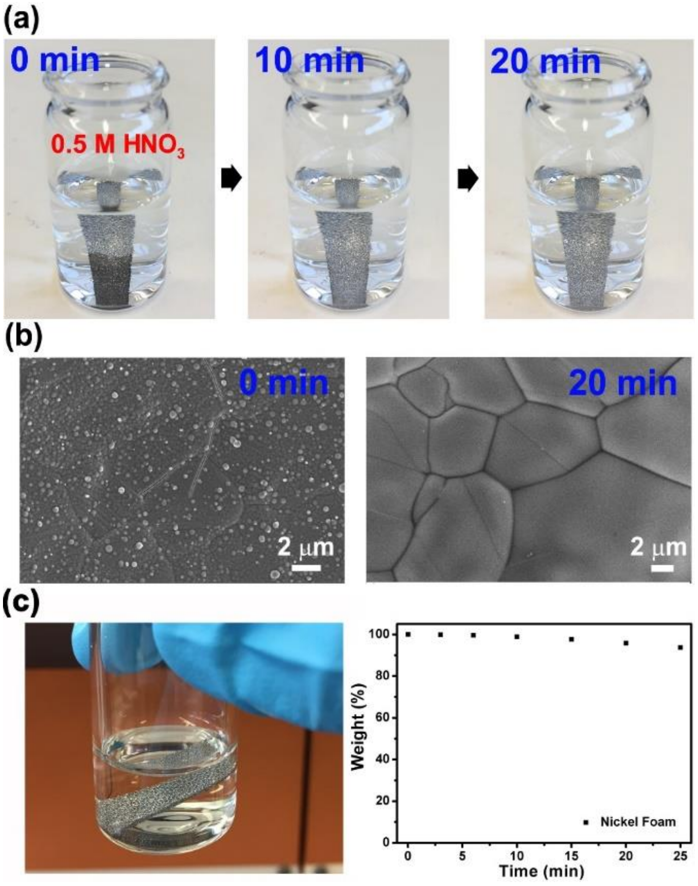

Fig. S18 a Photographs of NiO/NiNDs@NF immersed in 0.5 M HNO_3 solution. **b** SEM images of NiO/NiNDs@NF before (0 min) and after (20 min) treating with 0.5 M HNO_3 solution. **c** Photograph of NF immersed in 0.5 M HNO_3 solution and the change of weight with respect to time

Table S1 Comparation of the overpotential behavior of modified NF

Catalyst	Water electrolysis test	Electrolyte Solution	Current Density (*j*; mA cm^{-2})	Overpotential at the corresponding *j* (mV)	References
NiO/NiNDs@NF	HER	1 M KOH	10	120	This work
			20	156	
	OER	1 M KOH	20	320	
			50	358	
NiS@NF	HER	1 M KOH	20	158	[S2]
	OER	1 M KOH	50	335	
NiFe LDH@NF	HER	1 M KOH	20	251	[S3]
	OER	1 M KOH	50	349	
Ni_3S_2@NF	HER	1 M KOH	10	223	[S4]
	HER	1 M KOH	20	292	
	OER	1 M KOH	10	260	
NiSe@NF	HER	1 M KOH	10	96	[S5]
	OER	1 M KOH	20	270	
$Ni(OH)_2$@NF	HER	1 M KOH	10	250	[S3]
	OER	1 M KOH	20	ca. 372	
Ni_3N@NF	HER	1 M KOH	10	121	[S6]
			20	177	

Supplementary References

[S1] C.M. Cardona, W. Li, A.E. Kaifer, D. Stockdale, G.C. Bazan, Electrochemical considerations for determining absolute frontier orbital energy levels of conjugated polymers for solar cell applications. Adv. Mater. **23**(20), 2367-2371 (2011). https://doi.org/10.1002/adma.201004554

[S2] W. Zhu, X. Yue, W. Zhang, S. Yu, J. Wang, Nickel sulfide microsphere film on ni foam as an efficient bifunctional electrocatalyst for overall water splitting. Chem. Commun. **52**, 1486-1489 (2015). http://doi.org/10.1039/C5CC08064A

[S3] J. Luo, J.H. Im, M.T. Mayer, M. Schreier, M.K. Nazeeruddin et al., Water photolysis at 12.3% efficiency via perovskite photovoltaics and earth-abundant catalysts. Science **345**(6204), 1593-1596 (2014). https://doi.org/10.1126/science.1258307

[S4] L. Feng, G. Yu, Y. Wu, G. Li, H. Li et al. High-index faceted Ni_3S_2 nanosheet arrays as highly active and ultrastable electrocatalysts for water splitting. J. Am. Chem. Soc. **137**, 14023-14026 (2015). https://doi.org/10.1021/jacs.5b08186

[S5] C. Tang, N. Cheng, Z. Pu, W. Xing, X. Sun, NiSe nanowire film supported on nickel foam: an efficient and stable 3D bifunctional electrode for full water splitting. Angew. Chem. Int. Ed. **54**(32), 9351-9355 (2015). https://doi.org/10.1002/anie.201503407

[S6] Z. Xing, Q. Li, D. Wang, X. Yang, X. Sun, Self-supported nickel nitride as an efficient high-performance three-dimensional cathode for the alkaline hydrogen evolution reaction. Electrochim. Acta **191**, 841-845 (2016). https://doi.org/10.1016/j.electacta.2015.12.174

Movie S1 Electrodeposition process of NiND clusters

Movie S2 Water electrolysis of large-sized NiO/NiNDs@NF electrodes in 1 M KOH ($S_{electrodes}$～70 cm^2; distance between electrodes is ～2 cm). Current density is 13 mA cm^{-2}
